# The intestinal microbiome of fish under starvation

**DOI:** 10.1186/1471-2164-15-266

**Published:** 2014-04-05

**Authors:** Jun Hong Xia, Grace Lin, Gui Hong Fu, Zi Yi Wan, May Lee, Le Wang, Xiao Jun Liu, Gen Hua Yue

**Affiliations:** 1 Molecular Population Genetics Group, Temasek Life Sciences Laboratory, 1 Research Link, National University of Singapore, Singapore 117604, Republic of Singapore; 2 Department of Biological Sciences, National University of Singapore, 14 Science Drive 4, Singapore 117543, Singapore

**Keywords:** Fish, Microbiome, Starvation, Stress, Interaction, Nutrition

## Abstract

**Background:**

Starvation not only affects the nutritional and health status of the animals, but also the microbial composition in the host’s intestine. Next-generation sequencing provides a unique opportunity to explore gut microbial communities and their interactions with hosts. However, studies on gut microbiomes have been conducted predominantly in humans and land animals. Not much is known on gut microbiomes of aquatic animals and their changes under changing environmental conditions. To address this shortcoming, we determined the microbial gene catalogue, and investigated changes in the microbial composition and host-microbe interactions in the intestine of Asian seabass in response to starvation.

**Results:**

We found 33 phyla, 66 classes, 130 orders and 278 families in the intestinal microbiome. Proteobacteria (48.8%), Firmicutes (15.3%) and Bacteroidetes (8.2%) were the three most abundant bacteria taxa. Comparative analyses of the microbiome revealed shifts in bacteria communities, with dramatic enrichment of Bacteroidetes, but significant depletion of Betaproteobacteria in starved intestines. In addition, significant differences in clusters of orthologous groups (COG) functional categories and orthologous groups were observed. Genes related to antibiotic activity in the microbiome were significantly enriched in response to starvation, and host genes related to the immune response were generally up-regulated.

**Conclusions:**

This study provides the first insights into the fish intestinal microbiome and its changes under starvation. Further detailed study on interactions between intestinal microbiomes and hosts under dynamic conditions will shed new light on how the hosts and microbes respond to the changing environment.

## Background

Wildlife species can often be affected by starvation due to changes in environmental factors (e.g., temperatures, salinity and oxygen concentration). Starvation not only affects the nutritional and health status of animals, but also the microbes in their intestines [1]. Physiological changes during starvation drives the animals and their intestinal microbes to rapidly acclimate to the situation [1-3].

The composition and interactions of the gut microbiota may affect the amount of energy extracted from the diet and energy harvest [4,5], and play an important role in the metabolism of dietary substrates and immune system modulation [6]. The balance of gut microbial community composition can be altered by many factors, including stress [7,8], antibiotic exposure [9], nutritional status [10], age [11], degree of hygiene [12] and bacterial infection [13]. Diets play a dominant role in shaping gut microbiota and altering the metabolism and population sizes of key symbiont species, resulting in biological changes to the host [14]. An altered microbiota in the intestine can lead to altered immune functions of hosts, and also increase the risk of disease [14,15]. However, as studies on gut microbiomes have largely been performed on humans and land animals [16-21], not much is known on gut microbiomes and their changes under changing environmental conditions in organisms living in aquatic habitats.

Studies of bacteria community are traditionally carried out on the basis of representative genomes and signature genes such as 16S ribosomal RNA (rRNA). However, analyses of 16S rRNA can only appraise the phylogenetic composition of a sample and provide no direct information about its functional capabilities [22]. Full scale metagenomics can augment the information content of the data generated by not only determining the relative abundance of all genes, but a description of the functional potential of each community as well. Compared with the signature gene-based methods, this new technique gives a much broader description than phylogenetic surveys [23]. Recently, an analysis of 16S rRNA was performed to assess the bacteria composition in the grass carp [24,25] and the zebrafish [26]. However, little is known about the gene content of fish microbiota and the effects of starvation on microbial populations in fish.

The Asian seabass *Lates calcarifer* is an important farmed foodfish species. The fish has the ability to tolerate all levels of salinity from fresh to seawater allowing them to be cultured in both environments. This species feeds on crustaceans, mollusks, and smaller fish [27]. In this study, we sequenced the metagenomic DNA isolated from the intestine of the Asian seabass using a Hiseq 2000 sequencer. We characterized the intestinal microbiome, and analyzed the influence of starvation on the composition of the gut microbial communities. By using comparative metagenomic studies and analyzing expression of selected host genes with quantitative reverse-transcriptase PCR (qRT-PCR), we have constructed a primary microbial gene catalogue, and investigated the changes of the microbial composition and the host-microbe interactions in the intestine under starvation. Our analysis suggests variable microbiomes and host-microbiota signatures in the fish intestines in response to starvation.

## Results and discussion

### Metagenomic analysis suggests variable microbiomes in fed vs. starved seabass intestines

The intestine is one of the major organs in fish that interacts with the environment, and is involved in adaptations and stress responses. The intestinal microbiota are composed of a diverse and vast population of microorganisms [28]. To characterize and compare the microbial communities in the Asian seabass intestine in response to starvation, we sequenced two metagenomic DNA samples isolated from Asian seabass intestines at eight days post fasting challenge (experimental sample) and from fed seabass intestines (control) by using the Illumina Hiseq 2000 sequencing system. The sequencing yielded 71,254,936 reads for the experimental sample and 64,649,316 reads for the control sample. The sequences gave an overall average length of 101 bp and represented 13.7 Gb of DNA data. After trimming of the low-quality sequences and adaptors, 69,893,230 and 62,408,866 high-quality reads for the experimental sample and the control sample were obtained. The high quality reads were classified from phylum to family using the program MetaPhlAn [29] with the default settings.

**Figure 1:**
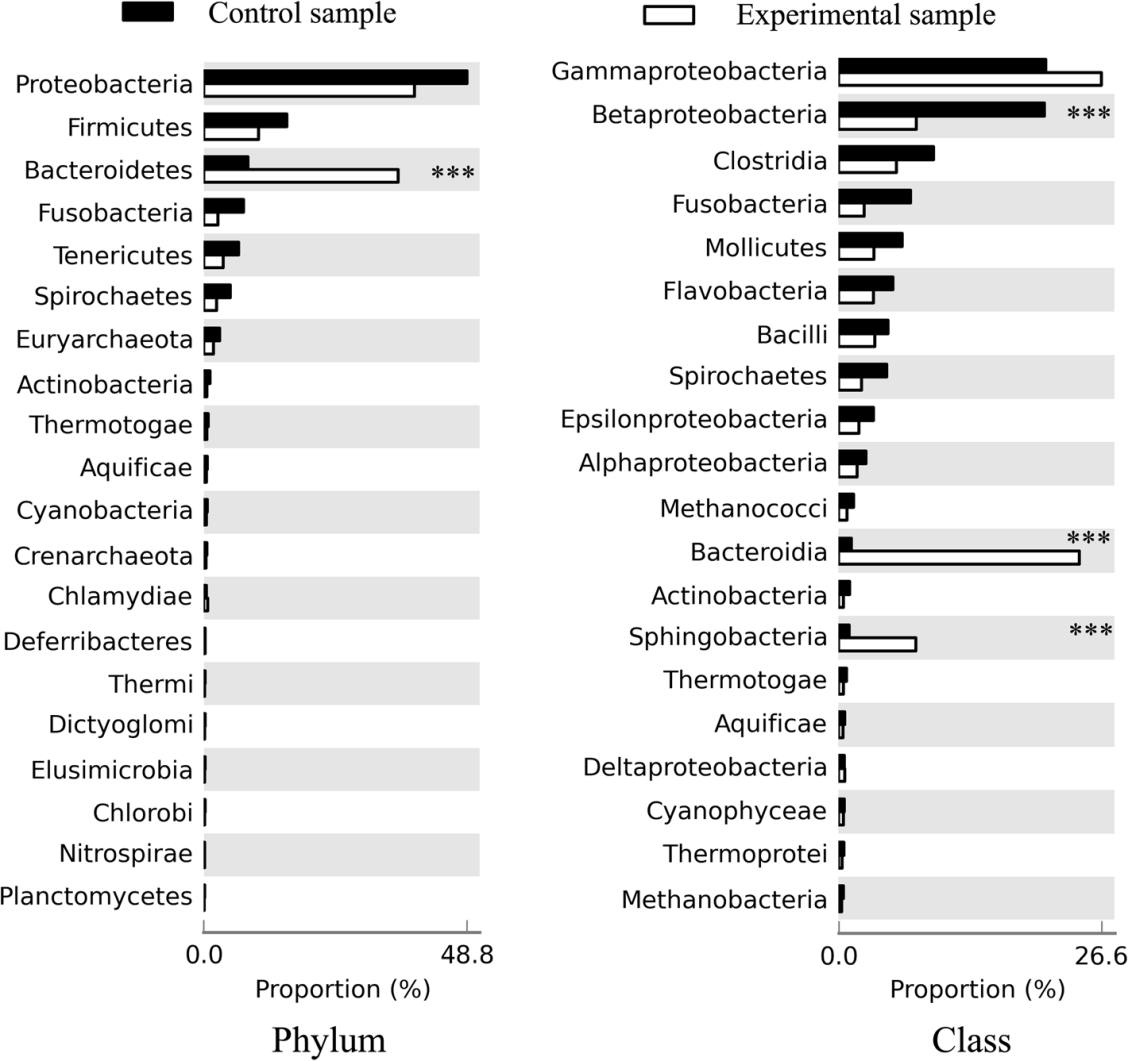
**Comparison of the taxonomic composition in the intestinal microbiome of Asian seabass in response to starvation.** The relative abundances (percentage) for the top 20 taxa of the metagenomes at phylum and class levels between the control sample (Feed) and the experimental sample (Fast) are presented. Asterisks indicate significant differences (Bootstrap test: ***P < 0.001).

In the control sample, we found that 96.3% of the metagenomes were assigned to bacteria and 3.7% were assigned to Archaea. The metagenome included 33 phyla, 66 classes, 130 orders and 278 families (Additional file 1). Proteobacteria (48.8%), Firmicutes (15.3%), Bacteroidetes (8.2%) and Fusobacteria (7.3%) were the four most abundant bacteria phyla (Figure 1). The results were generally consistent with those observed in the intestinal samples of other fish species. In grass carp, Proteobacteria and Firmicutes were dominant in the gut bacteria [25], and in adult zebrafish, Proteobacteria (79.4%) and Fusobacteria (13.6%) phyla were common members of the intestinal microbiota [26]. However, there are some differences in the intestinal microbiota among different fish species. For example, in grass carp, Actinobacteria (more than 10%) were the most prevalent members of the intestinal bacterial communities, and they were more abundant than Bacteriodetes [25]. In our study, only 1.1% and 0.5% of the microbiota members were Actinobacteria in the control sample and in the experimental sample, respectively. Therefore, Actinobacteria were much less abundant than Bacteriodetes (8.2%) in the Asian seabass intestine. Diet plays a dominant role in shaping gut microbiota and changing key populations [14,30,31]. The grass carp is an herbivorous species, while Asian seabass is a strict carnivore, feeding on crustaceans, mollusks and smaller fish in the wild. Therefore, the difference in bacterial communities between grass carp and Asian seabass may be caused by evolved differences that have arisen in the context of differing diets. In addition, our data supported prior findings suggesting that gut microbiota differ between fish and mammalian intestines. Firmicutes and Bacteroidetes were the most dominant phyla in mammals [32,33], whereas Proteobacteria were the most abundant bacteria phyla in fish. This difference suggests that gut environments differ in their selectivity/hospitability for bacterial proliferation.

In the experimental sample, we found that 97.7% of the metagenomes were assigned to bacteria, and 2.3% were assigned to Archaea (Additional file 1). Proteobacteria (39.1%), Bacteroidetes (36.0%) and Firmicutes (10.1%) were the three most abundant bacterial species in the experimental sample (Figure 1). Both metagenomes showed very small differences in diversity when reviewed in light of the numbers of present taxa (Additional file 1). However, dramatic differences in microbial community composition of the fish intestine across samples were observed (Figure 1 and Table 1). We found that a total of three orders and four families showed apparent shifts in relative abundance in response to starvation. More detailed information on these shifts in microbial community composition was presented in Additional file 1. These findings are consistent with previous studies in humans, in which the relative abundances of different bacterial species in the gut microbiota were highly sensitive to diet [10,32].

**Table 1:** Shift in intestinal microbiota in Asian seabass in response to starvation

**Rank**	**Classification**	**Control (%)**	**Experimental (%)**	***P*-value**
Phylum	Bacteroidetes	8.2	36.0	<1E-15
Class	Betaproteobacteria	20.8	7.9	<1E-15
Class	Bacteroidia	1.3	24.4	<1E-15
Class	Sphingobacteria	1.1	7.8	<1E-15
Order	Burkholderiales	20.3	7.4	<1E-15
Order	Bacteroidales	1.3	24.4	<1E-15
Order	Sphingobacteriales	1.1	7.8	<1E-15
Family	Oxalobacteraceae	20.0	7.1	<1E-15
Family	Sphingobacteriaceae	1.1	7.8	<1E-15
Family	Bacteroidaceae	0.6	23.2	<1E-15
Family	Aeromonadaceae	0.1	2.4	<1E-15

The significant changes in microbial composition (relative abundance, %) from family to phylum in the fish intestines in response to starvation are presented. *P*-value indicates significance level as calculated using a bootstrap statistical test.

Significantly more reads were assigned to the Bacteroidetes phylum in the experimental sample (36%) as compared to the control sample (8.2%). At the class level, Bacteroidia (1.3% in the control sample vs. 24.4% in the experimental sample) and Sphingobacteria (1.1% in the control sample vs. 7.8% in the experimental sample) contributed to higher percentages of the microbiota in the experimental sample than in the control sample. The significant elevation of Bacteroidetes in the intestinal community of the starved seabass sample is in agreement with some other studies [2,34,35] on dietary shifts. For example, in mice [34], fasting was associated with a significant increase in the proportional representation of the Bacteroidetes [from 20.6% (fed) to 42.3% (fasted)]. Bacteroides with a much larger genome size (e.g., *Bacteroides fragilis* Strain NCTC9343: 5,205,140 bp) are normally mutualistic in the animal gastrointestinal flora. A large part of the proteins made by the Bacteroides genome are able to break down polysaccharides and metabolize their sugars [36]. They play a fundamental role in the processing of complex molecules to simpler ones in the host intestine. Their ability to harvest alternative energy sources from food might allow Bacteroides to be more competitive than other bacteria in the fish intestine during starvation.

In contrast, Firmicutes are more abundant in the control sample (15.3%) than in the experiment sample (10.1%). The pattern that the relative abundance of Firmicutes in the gut is positively correlated with dietary caloric intake is frequently observed in humans, mice, pythons and in zebrafish [37,38]. In addition, the Betaproteobacteria class was more abundant with 20.8% in the control sample as compared with 7.9% in the experimental sample. The increased abundance of Betaproteobacteria class in response to feed was also observed in zebrafish [39], suggesting that Betaproteobacteria play a role in the interactions between microbiota, diet and hosts in fish.

To better understand the temporal changes of microbial community composition during starvation, we further amplified the 16S rRNA sequences from metagenomic DNA that was isolated directly from the intestine samples at three, six and twelve days post fast by using conserved primers targeting the domain Bacteria, and generated six libraries (one library per treatment per time) for Sanger sequencing. For each time point of the fasting challenges, around 350 high-quality sequences with a minimum length of 500 bp were obtained. The taxonomic classification for the control library d12 (fed fishes) is drastically different from the control libraries d3 and d6 that were composed entirely of Cetobacterium. This is unexpected and may suggest that the classification is invalid at lower level (e.g., species and genus) due to a limitation of the BLAST-based approach. However, the phylogenetic analysis of the 16S rRNA gene data suggests a similar shift of bacteria components post the fasting challenge as our next generation sequencing-based metagenomic study (Additional file 2). For example, Bacteroidales were the most dominant order in the experimental samples, but were negligible in the control samples; Proteobacteria phylum (including Yersinia, Pectobacterium, Acinetobacter) and Fusobacteria phylum (including Cetobacterium) were the most dominant components in the control samples, but only low proportions of these bacteria were detected in the experimental samples at the three time points. Our data showed that the microbial community composition in the fish intestines can be quickly changed in response to starvation in less than three days. However, in human, a 10-day dietary intervention is not sufficient to alter the enterotype of an individual [40]. Compared to the changes of microbial composition in humans, the changes of the intestinal microbiota in fish are more rapid and significant. A recent comparison of the microbiota of zebrafish intestines and their housing water under fed and starved conditions suggested that some bacterial taxa observed in the fish intestine are found at similar frequency in the water, while other taxa are enriched specifically in the intestine [39]. This suggests that the fish gut looks a lot more like the surrounding environment, and even more so under starvation conditions due to a limited selective environment as compared to mammals.

### Metagenomic comparisons between fed and starved fishes by functional investigations

Functional investigations by blasting the metagenomic data against public databases may reveal the genetic determinants of microbial interactions, and illuminate the mechanisms responsible for directing the changes in microbial diversity in response to fasting challenges. In order to generate a primary catalogue of microbial genes from the fish intestines, and explore the data differences caused from the fasting challenge, we first performed de novo assembly for the high quality reads. The de novo assembly of the high quality reads from two samples produced 326,789 scaffold sequences with a N50 length of 1,447 bp. Eighty-three percent of these reads was mapped back to the assembly. Of the scaffolding sequences, 299,018 sequences had a minimum length of 500 bp and gave a total length of 443 Mb with a N50 length of 1,857 bp. Sixty-one percent of the reads from the control sample and sixty-nine percent of the reads from the experimental sample were mapped back to the assembled sequences (≥ 500 bp). We then used the software Prodigal (Prokaryotic Dynamic Programming Gene finding Algorithm v2.15) [41] to predict the whole range of open reading frames (ORFs) for the metagenomic DNA sequences (≥ 500 bp). Even though a total of 462,828 ORFs were found, most of the ORFs appeared to be incomplete. We classified these predicted genes by aligning them to the Clusters of Orthologous Groups (COG) protein database that was derived from all of the proteins encoded by the genomes of bacteria, archaea and unicellular eukaryotes [42]. We found that 28,989 predicted ORFs were assigned to 3,182 COG orthologous groups, which could be classified into 30 COG functional categories (Additional files 3 and 4). Nearly six percent of the reads from the control sample and thirteen percent of the reads from the experimental sample were mapped back to the predicted ORF sequences with COG annotation.

**Figure 2:**
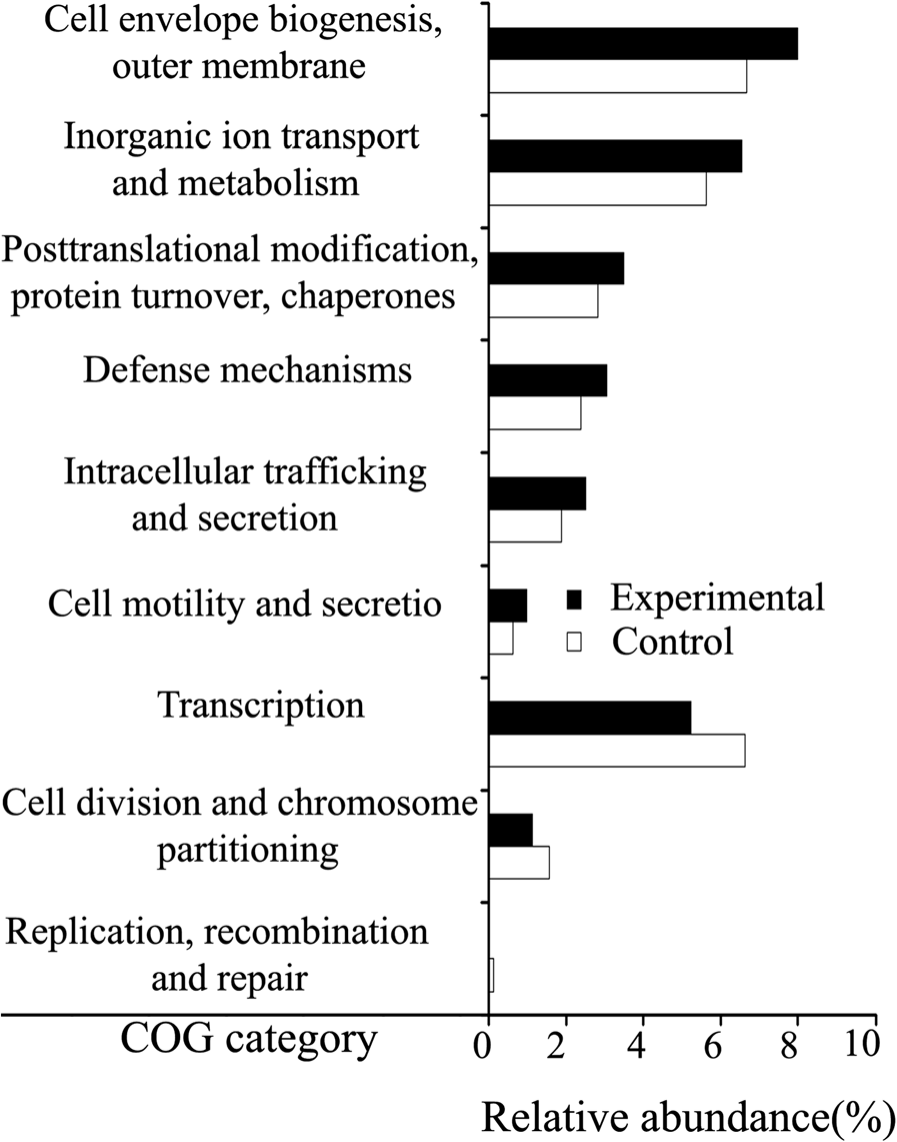
**Comparisons of COG functional categories significantly enriched or depleted in the Asian seabass intestinal microbiomes.** The relative abundances (percentage) for nine significant different COG functional categories (*P* < 1E-15) in response to fast challenge are presented. ‘Fast’ shows the experimental samples without feeding and ‘Feed’ shows the control samples that were given feed.

There were significant differences in read counts of COG functional categories between the two datasets (Additional file 4). We found that three functional categories, including transcription, cell division and chromosome partitioning and replication, recombination and repair were significantly depleted in the experimental samples (*P* < 1E-15). Six functional categories, e.g., cell envelope biogenesis, outer membrane and defense mechanisms were significantly enriched during starvation (*P* < 1E-15; Figure 2).

To further identify genes in the intestinal microbiome, which are associated with starvation, statistical comparisons for the 3,182 COG orthologous groups were performed. Substantial fluctuations in the metagenomic components were found during starvation. We observed that 44 orthologous groups were significantly depleted (Additional file 5). These included the genes CitF (Citrate lyase, alpha subunit), EntF (Non-ribosomal peptide synthetase modules and related proteins) and transketolase (N-terminal subunit). These genes were mainly associated with energy production and conversion, secondary metabolites biosynthesis, transport, and catabolism, and carbohydrate transport and metabolism. Thirty genes were significantly enriched, including RhsA (Rhs family protein), NanH (Neuraminidase) and TesA (Lysophospholipase L1 and related esterases). Of the enriched genes, 16 (53%) were associated with functional categories including Carbohydrate transport and metabolism, Inorganic ion transport and metabolism, and Amino acid transport and metabolism.

Gut microbiota provide their host with a physical barrier to pathogen infection by competitive exclusion and production of antimicrobial substances [43]. Antibiotics are produced by bacteria to outcompete intestinal pathogens. To further explore the microbe-microbe interactions, we downloaded 19,080 prokaryote genes related to antibiotic activity from UniProt protein database (http://www.uniprot.org/). By using local blast alignment, 1,674 of the metagenomic sequences were mapped to 300 antibiotic related genes (Additional file 6). We found that seventy-two genes (e.g., ABC transporter related protein, dTDP-glucose 4,6-dehydratase, glucose-1-phosphate thymidylyltransferase, isoleucine-tRNA ligase 2) were significantly depleted in the experimental sample. Also seventy-two genes (e.g., efflux pump membrane transporter BepE, ribosomal RNA large subunit methyltransferase N) were significantly overrepresented in the experimental sample (Additional file 7). The overall mapping rate was only 1.67% in the control sample, while in the experimental sample, the rate was higher (9.42%) (a ratio of 5.6 between two datasets). As compared with the overall mapping rate of the reads to COG orthologous groups (a ratio of 2 between the two datasets), a much higher proportion of reads in the experimental sample mapped to antibiotic related genes. Our data suggest that the relative proportions of bacteria with genes related to antibiotic activity may be increased in the fish intestine in response to starvation. Of the enriched genes, at least six genes were related to beta-lactamase activity, e.g., beta-lactamase type II, putative beta-lactamase hcpD, putative beta-lactamase hcpE and beta-lactamase blaTLA-1. An increase in antibiotic-producing strains could benefit the fish by excluding potential pathogens from colonizing the intestines. This observation could be due to the reduction in overall bacterial abundance with the exception of a few larger genome generalists (in Bacteroidetes) that harbor antibiotic resistance genes and can, in the conditions of nutrient deprivation, turn to harvesting host-produced products. This is in agreement to the human intestine-adapted bacterial symbiont, which turns to host mucus glycans when polysaccharides are absent from the diet [44]. However, it is also possible that these genes may simply be hitching a ride with a microbe that is a generalist which can better survive these conditions.

### Functional interactions between intestinal microbiota and their hosts during starvation

**Figure 3:**
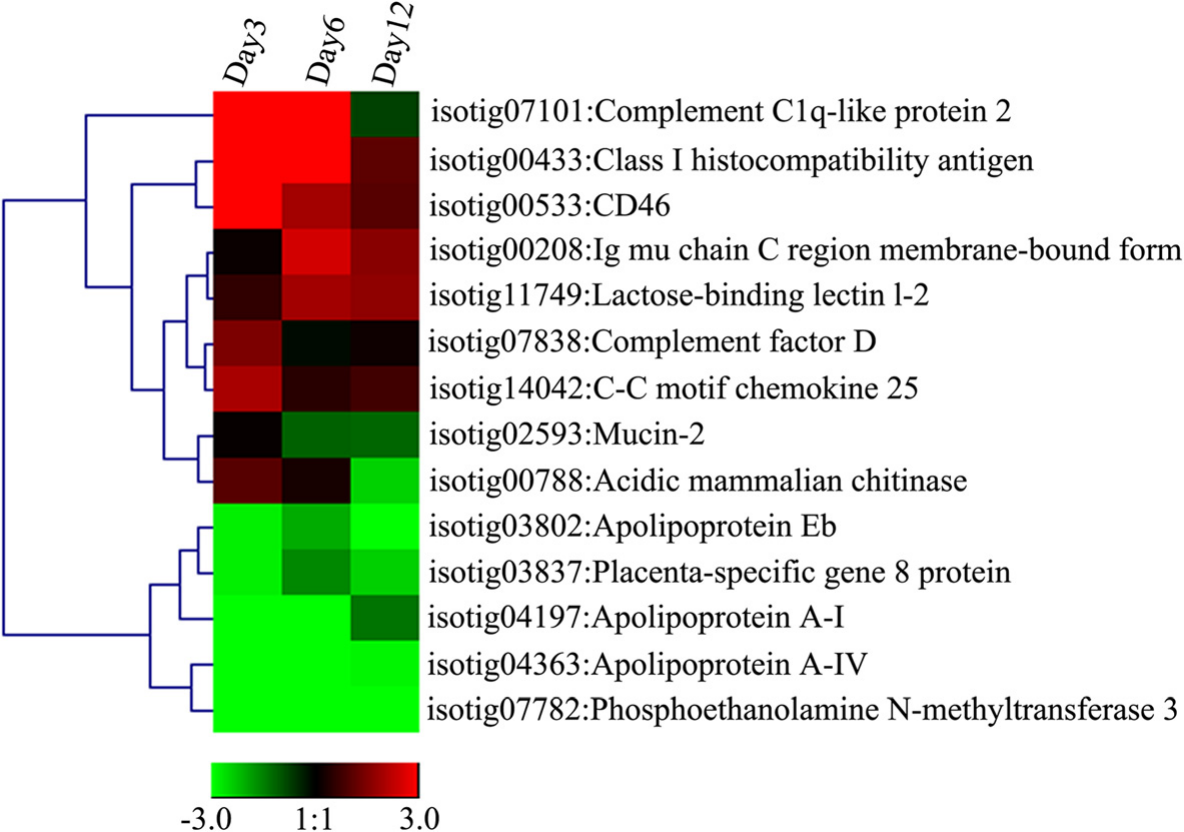
**Gene expression profiles in the Asian seabass intestines across starvation treatments as revealed by qPCR.** A heat map for fold changes of gene expression in 14 genes at three, six, twelve days post fast in the Asian seabass intestine is shown. Red indicates increased expression and green shows decreased expression in response to fast challenge.

The importance of the intestinal microbiome in the development of both the intestinal mucosal and systemic immune systems have been shown in mammals [45]. In most species of fish, starvation is experienced during certain periods of every year largely due to environmental conditions [46,47]. Starvation affects many physiological changes to satisfy its energy requirements in fish [47]. Recent studies have shown that starvation can induce stress responses in fish. For example, starvation resulted in a significant reduction of the intestine length, the surface area of the intestinal mucosa and the mucosal thickness [48,49] and increased xenobiotic resistance and paracellular permeability of epithelial cells in the anterior intestine [50]. To further explore the host-microbe interactions in fish intestine, ten genes related to immunity, defense and inflammatory response and four genes related to lipid metabolism and transport, and cholesterol metabolism were selected for expression analysis (Additional file 8). Temporal expression of the 14 genes was analyzed by qRT-PCR using RNA isolated from the Asian seabass intestine sampled at three, six and twelve days post fasting. The expression analysis clustered these genes into two groups (Figure 3). The apolipoprotein and phosphoethanolamine N-methyltransferase 3 genes related to lipid metabolism and transport, cholesterol metabolism [51,52], and the placenta-specific gene 8 protein gene showing defense response to bacteria [53] were down-regulated in the starved experimental fish at all time-points of fasting (2.6 - 61.1 fold). Interestingly, mucin-2, which provides an insoluble mucous barrier that serves to protect the intestinal epithelium [54], was weakly increased at three days post fasting (1.1 fold), but quickly down-regulated at six and twelve days post fasting (2.2 - 2.3 fold). The intestinal barrier functions as one of the first lines of defense against microbial pathogens [55]. Dramatic decreases in the gene expression of mucin-2 and placenta-specific gene 8 protein may lead to disruption of the mucosal barrier in the long term, increasing susceptibility to pathogen infections. Moreover, the immune-related genes, such as complement C1q-like protein 2 (52–270 fold), class I histocompatibility antigen (20–22 fold) and CD46 (4–15 fold) were highly up-regulated from samples at three and six days post fast. However, at 12 days post fasting, immune related genes were only slightly increased, e.g., class I histocompatibility antigen (2 fold) and CD46 (2 fold), or down-regulated, e.g., complement C1q-like protein 2 (1.7 fold). These data suggest that long-term malnutrition or starvation will damage the mucosal barrier of the Asian seabass by increasing the permeability of the intestinal mucosal barrier. The intestinal microflora, especially the opportunistic pathogens could then cross the intestinal barrier when the intestinal mucosal barrier is damaged or the normal flora has been destroyed by antibiotics or nutrition deficiency. Based on prior findings from mammals, short-term stress experienced at the time of immune activation can enhance innate and adaptive immune responses, but long-term stress can suppress immunity by decreasing immune cell numbers and function and/or increasing active immunosuppressive mechanisms [56]. However, the underlying mechanisms of the nexus between host immune system and intestinal microbiota in response to nutrient changes need further exploration. Future studies targeting the microbial metaproteome and the interactions between the intestinal microbiota and their hosts in response to different nutrition conditions or stressors with a combination of continued sequencing, cultivation and functional genomics are likely to provide further insights into the functions of metagenomes in the fish intestine.

## Conclusions

We determined the primary microbial gene catalogue and investigated the changes of the microbial composition and host gene expression in the intestine of Asian seabass in response to starvation. Proteobacteria (48.8%), Firmicutes (15.3%) and Bacteroidetes (8.2%) were the three most abundant bacteria phyla. The components of intestinal microbiota community shifted in response to starvation with significant enrichment of Bacteroidetes, but depletion of Betaproteobacteria. There were significant differences in COG functional categories and orthologous groups, and genes related to antibiotic activity in response to starvation. Genes related to antibiotic activity in the microbiome were significantly enriched in response to starvation, and host genes related to immune system were generally up-regulated. This study provides the first insights into the intestinal microbiome and its changes under starvation in fish. Since our next generation sequencing datasets are from two pooled samples, the inter-individual variation can not be evaluated, which strongly influenced the power of our data analysis. Further detailed study on interactions between individual intestinal microbiomes and hosts under dynamic conditions will shed new light on how the hosts and microbes respond to the changing environment.

## Methods

### Ethics statement

All handling of fishes was conducted in accordance with the guidelines on the care and use of animals for scientific purposes set up by the Institutional Animal Care and Use Committee (IACUC) of the Temasek Life Sciences Laboratory, Singapore. The IACUC has specially approved this study within the project “Breeding of Asian seabass” (approval number is TLL (F)-12-004).

### Fish management, fasting challenge, sample collection and metagenomic DNA extraction

An Asian seabass population was constructed by mass crossing of 50 F1 generation Asian seabass in the Marine Aquaculture Center, Singapore. The fingerlings were transferred to the animal facility of the Temasek Life Sciences Laboratory, Singapore. By gradually diminishing the salinity of the water, the fish was accustomed to living in freshwater. For challenge experiments, twelve fishes at the age of 11 months (~330 g) were transferred equally to two 1,000 litre recirculating tanks with water volume of ~500 litres in each tank. Nearly 30% water was changed per day. Six fishes in the control tank were fed to satiation twice daily with pelleted feed (Biomar, Nersac, FR, France) that was kept in a cold room. The uneaten feed was removed from the bottom of the tank. The other six fishes in the test tank were not given access to feed before sampling. All fishes were euthanized by immersion in ice-water (4°C or less) for nearly 20 minutes at 8 days post fasting. Deceased fish were then decontaminated with 70% ethanol and transferred into a laminar flow hood. Entire intestines were removed from each fish of each tank after dissection under sterile conditions. To isolate microbial cells, the pooled whole intestine from the fishes in the same tank were cut into small pieces and homogenized in ~10-fold dilution of cold sterile 1× phosphate-buffered saline solution using autoclaved mortars and pestles, which made it possible to isolate most of the gut wall-associated microbes. The mixtures were centrifuged at low speed, and the suspensions were filtered with a 100 μm Nylon net filter (Millipore, Billerica, MA, USA). The filtrate was centrifuged at full speed for 20 min. The cells were collected for isolation of metagenomic DNA using the QIAamp DNA Stool Mini Kit (Qiagen, Valencia, CA, USA) according to the manufacturer’s instructions. DNA obtained was submitted to Macrogen Inc. (South Korea) for 100-cycle paired-end sequencing with the Illumina Hiseq 2000 sequencing system (Illumina, San Diego, CA, USA).

### Fasting and sampling of tissues for quantitative real-time PCR (qPCR) and 16S rRNA sequencing

Thirty-six Asian seabass at the age of three months (body weight: ~50 g) originally maintained in a 1,000 L tank containing 500 L of freshwater were divided equally into two tanks containing 3,00 L of freshwater. Eighteen fishes, used as a control, were fed twice daily with pelleted feed (Biomar, Nersac, FR, France), and the other eighteen fishes in the test tank were not given access to feed before sampling. Six fishes from each group were sacrificed at three, six and twelve days post fasting, respectively. Before experiments, the pestles and dissecting tools were first soaked in 5% concentration of Clorox bleach (The Clorox Company, Oakland, USA) for ~15 minutes, and autoclaved for 20 minutes after washing and rinsing in ddH2O. The working surface area and the deceased fish were decontaminated with 70% ethanol. Small sections (~1 cm of length) from the middle part of the intestine samples were taken and homogenized in 1 ml of Trizol reagent (Invitrogen, Carlsbad, USA) with sterile KONTES® pellet pestle driven by a cordless motor (Fisher Scientific, New Hampshire, USA). RNA isolation was conducted using the Trizol reagent (Invitrogen, Carlsbad, USA) according to the manufacturer’s instructions. The remaining intestine samples from each fish was taken for DNA isolation using the QIAamp DNA Stool Mini Kit (Qiagen, Valencia, CA, USA) according to the manufacturer’s instructions.

### 16S rRNA amplification, cloning and sequencing

Bacterial 16S rRNAs were amplified by PCR using forward primer S-D-Bact-0008-a-S-20 (5′ AGA GTT TGA TCC TGG CTC AG 3′) [57], which targets the Bacteria domain, and the reverse primer S-*-Univ-1492-b-A-21 (5′ ACG GCT ACC TTG TTA CGA CTT 3′) [58], which targets all living organisms. PCR was conducted as described in Suau et al. [59]. The purified products were ligated into the pGEM®-T Vector Systems (Promega, Madison, USA) and then transformed into *E. coli* strain XL-1 (Stratagene, CA, USA). Randomly picked clones from the libraries were sequenced by using single pass sequencing using M13 forward sequencing primer (Promega, Madison, USA) with a 3730 xl DNA analyzer (Applied Biosystems, Foster city, CA).

### Analysis of seabass intestinal gene expression with qPCR

From published transcriptome datasets of the Asian seabass [60-62], 14 genes related to immune and metabolism activity were selected for analysis of gene expressions in seabass intestines by qPCR (Additional file 8). One primer pair for each gene was designed using the program PrimerSelect (DNASTAR, Wilmington, DE, USA). Gene expression was analyzed as described in Xia et al. [61]. Briefly, equal aliquots of total RNA from each of the six fish intestines under the same conditions were pooled, and around 1 μg of the DNase I-treated total RNA were reverse transcribed to cDNA by MMLV reverse transcriptase (Promega, Madison, Wisconsin, USA) with random hexamer primers. PCR was performed in triplicate with the iQ SYBR Green Master Mix as described by the manufacturer in an iQ™5 Real Time PCR Detection System (Bio-Rad, Hercules, CA, USA). Ten-fold dilutions of the cDNA preparation were used as DNA templates. For the PCR reaction in a total volume of 20 μl, a master reaction mix (per tube) contains 10 μl of Supermix, 0.3 μl of forward primer (300 nM final), 0.3 μl of reverse primer (300 nM final), and 1 μl of the diluted cDNA template. Thermal cycling conditions were as follows: 3 min at 95°C, followed by 40 cycles: 95°C for 15 sec, annealing temperature for 30 sec, 72°C for 30 sec. The PCR products for each of the primer pairs giving one specific band were confirmed on 2% agarose gels stained with Ethidium Bromide fluorescence under ultraviolet light and by melting curve analysis (only one peak was observed). Elongation factor-1 alpha gene (EF1A) has been suggested as the reference gene in qRT-PCR assays [63,64]. For analysis of the change of gene expression, the values of triplicate qPCR reactions were normalized to EF1A gene expression, calculated by the ΔΔCT method. The normalized expression values were then used to construct a heat map by Cluster 3.0 with parameter settings as hierarchical clustering, uncentered correlation and complete linkage (http://bonsai.ims.u-tokyo.ac.jp/~mdehoon/software/cluster/software.htm#ctv).

### De novo assembly of the intestine metagenomes for the Asian seabass

Paired-end data were processed to filter the low quality reads and adaptors using the CLC Genomics Workbench (CLC bio, Cambridge, MA). De novo assembly of the high-quality short reads was carried out using the SOAPdenovo Assembler (V1.05) [65] with assembly parameters ‘-d -D -R -F -K 53’. After assembly, the resulting genomic sequences with a length less than 500 bp were filtered out with software NGS QC Toolkit [66]. The software packages Bowtie 2 [67] and SAMtools [68] were applied for aligning sequencing reads to the assembled genomic sequences and retrieving statistical data.

### Phylogenetic analysis, gene prediction and functional classification of the metagenomic data

MetaPhlAn (v1.7.3) is a computational tool for profiling the composition of microbial communities from metagenomic shotgun sequencing data by running BLAST searches against unique clade-specific marker genes identified from 3,000 reference genomes [29]. This tool was used for profiling the composition of microbial communities from our sequencing reads. The assembled genomic sequences were used to predict the whole range of ORFs with Prodigal (Prokaryotic Dynamic Programming Genefinding Algorithm v2.15) [41]. The predicted ORFs were then aligned to the protein database of Clusters of Orthologous Groups (COG) database [69] using BLASTp algorithm with an E-value threshold of E-7. Prokaryote protein gene sequences with the biological process category annotated as antibiotic resistance or antibiotic biosynthesis were downloaded from UniProt protein databases and used for gene functional analysis.

Taxonomic assignment for high quality 16S rRNA sequences was carried out using the Ribosomal Database Project (RDP; http://rdp.cme.msu.edu/) [70]. The rRNA analysis files downloaded from the RDP website were then imported into the software MEGAN [71] to analyze the taxonomic content of DNA reads.

### Identification of changed genes, pathways and taxonomic units in fed vs. starved gut microbiomes

The online tool Xipe (written by Beltran Rodriguez-Muller; http://edwards.sdsu.edu/cgi-bin/xipe.cgi) provides a non-parametric statistical analysis of the distribution of samples. This tool can be used to compare two different populations and identify the differences between those samples. The STAMP (**St**atistical **A**nalysis of **M**etagenomic **P**rofiles) [72] is a software package for analyzing metagenomic profiles. Both tools have been applied or suggested in previous metagenomic studies [73,74]. In our data analysis, the proportions of the relative abundance profiles of the composition of microbial communities exported from the software MetaPhlAn were first adjusted to read counts per million mapped. Bootstrap analyses of enrichment and depletion of bacterial taxa, genes and pathways between dietary treatments were carried out using the tool Xipe (with the parameters: sample size of 5,000 and confidence level of 0.9) and the software STAMP (with the following parameters: Bootstrap as statistical test, asymptotic as confidence interval method, Storey’s FDR as multiple test correction method and 10,000 bootstrap replicates). A feature is considered significantly different if it shows significant differences in both analyses.

### Availability of supporting data

The 16S rRNA sequences have been deposited in NCBI under accession #s: KC599554 - KC601631. The metagenomic sequence data are available under the DNA Data Bank of Japan (DDBJ) Sequence Read Archive database (accession no.: [DDBJ: DRR004449 and DRR004450]).

## Abbreviations

COG: Clusters of orthologous groups; rRNA: Ribosomal RNA; NGS: Next-generation sequencing; ORF: Open reading frame; qPCR: Quantitative real-time PCR; DDBJ: DNA Data Bank of Japan.

## Competing interests

The authors declare that they have no competing interests.

## Authors’ contributions

JHX performed the metagenomic data analysis. GL optimized the protocol for isolation of metagenomic DNA and extracted the DNA. FGH and XJL prepared the RNA samples, and WZY and ML performed gene expression by qRT-PCR. WL managed the fish. JHX and GHY conceived of and supervised the study. JHX wrote the manuscript. YGH finalized the manuscript. All authors read and approved the final manuscript.

## Supplementary Material

Additional file 1 Taxonomic profiles detected in the intestinal metagenomes of the Asian seabass. Click here for file

Additional file 2 **Comparison of the intestinal bacteria variation in Asian seabass as detected by 16S rRNA sequencing in response to starvation.** The generated 16S rRNA sequences for each sample are normalized to the total number of the sequences. Each unique color represents a sample. Each circle represents one taxon. The area size for each color within a circle is proportional to the relative abundance of one taxon in different samples. The samples at three, six, twelve days post fast are named as Fast-d3, −d6 and -d12, and the controls are named as Feed-d3, −d6 and -d12, respectively. Click here for file

Additional file 3 The information of significant annotation of the intestine metagenomes of the Asian seabass against COG database. Click here for file

Additional file 4 Comparison of COG functional categories of the intestinal metagenomes in the Asian seabass in response to starvation. Click here for file

Additional file 5 COG orthologus groups significantly enriched or depleted in the intestinal microbiome of the Asian seabass. Click here for file

Additional file 6 Genes in the Asian seabass intestinal microbiome involved in antibiotic activity. Click here for file

Additional file 7 Genes with antibiotic activity significantly enriched or depleted in the intestinal microbiome. Click here for file

Additional file 8 Primers used in the expression analysis of selected genes from the Asian seabass. Click here for file
