# Supplementary figures and images for: The intestinal microbiome of fish under starvation
Source: BMC Genomics. 2014 Apr 5;15:266. doi: 10.1186/1471-2164-15-266 (PMC4234480; doi:10.1186/1471-2164-15-266)

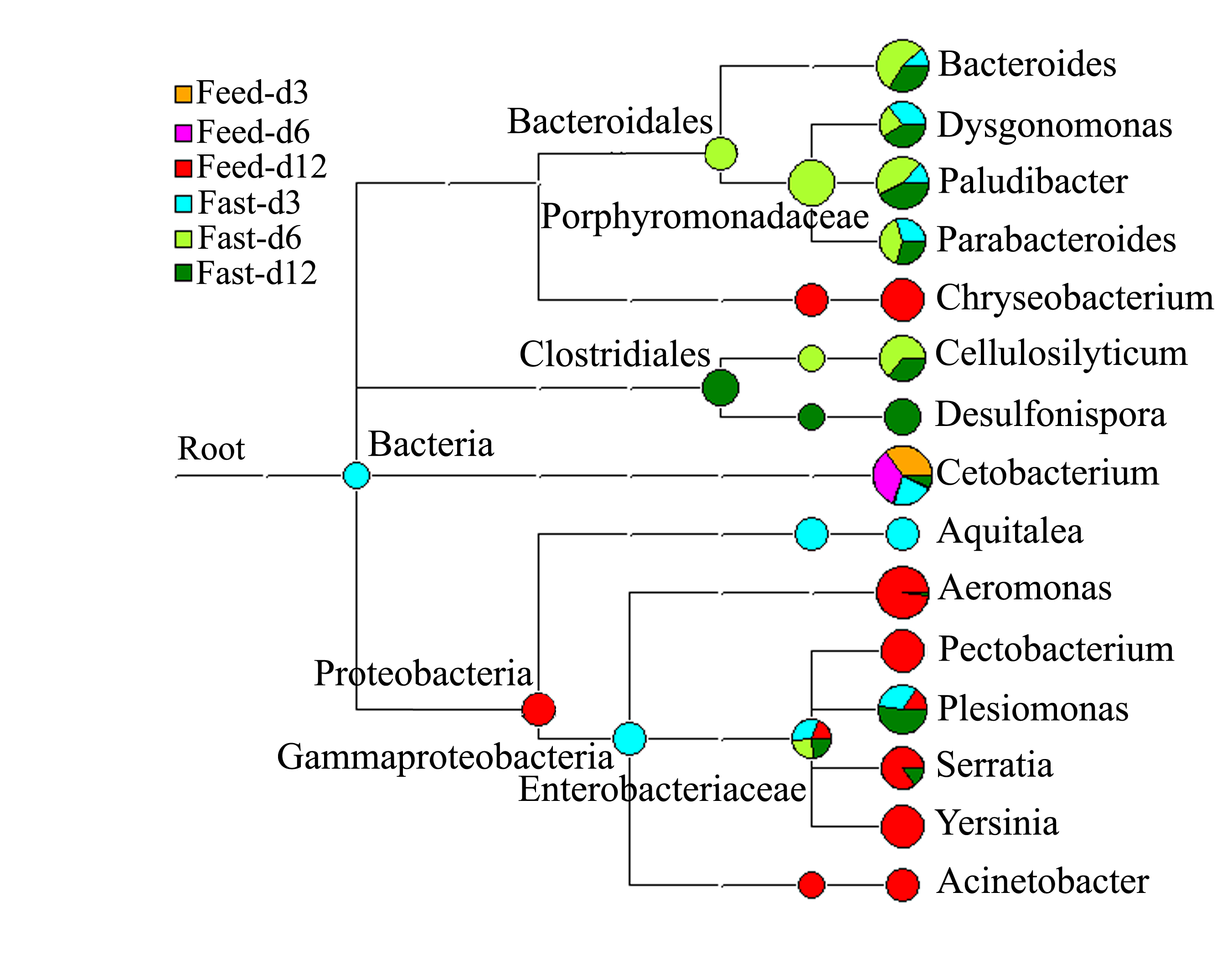

Supplement: Additional file 2 — Comparison of the intestinal bacteria variation in Asian seabass as detected by 16S rRNA sequencing in response to starvation. The generated 16S rRNA sequences for each sample are normalized to the total number of the sequences. Each unique color represents a sample. Each circle represents one taxon. The area size for each color within a circle is proportional to the relative abundance of one taxon in different samples. The samples at three, six, twelve days post fast are named as Fast-d3, −d6 and -d12, and the controls are named as Feed-d3, −d6 and -d12, respectively. [file 1471-2164-15-266-S2.tiff]
